# The ongoing indirect effect of the COVID-19 pandemic on a pediatric emergency department

**DOI:** 10.1371/journal.pone.0251003

**Published:** 2021-05-06

**Authors:** Matti Erlichman, Todd Zalut, Shepard Schwartz, Giora Weiser

**Affiliations:** 1 Shaare Zedek Medical Center and the Faculty of Medicine at the Hebrew University in Jerusalem, Pediatric Emergency Department, Jerusalem, Israel; 2 Emergency Department, Shaare Zedek Medical Center, Jerusalem, Israel; Technion - Israel Institute of Technology, ISRAEL

## Abstract

**Introduction:**

The SARS-CoV-2 coronavirus pandemic may cause significant morbidity and mortality in adults, yet severe cases are rare among children. The indirect impact of the pandemic on health care delivery in general and pediatric emergency department (PED) visits in particular has been widely reported.

**Aims:**

To assess the impact of the pandemic and the social restrictions imposed in its wake on PED visits and hospitalization rates in our Israeli medical center. We also sought to track these data in relation to the variation in pandemic severity and social restrictions over time. A comparison of this data with that of the adult emergency department was also performed.

**Methods:**

Data for this study were drawn from the Shaare Zedek Medical Center (SZMC), Jerusalem, Israel computerized databank. The daily number of PED and adult ER visits as well as hospitalizations resulting from these visits during the months January-July during the years 2018, 2019, 2020 were recorded. We compared the risk ratio for hospitalization in 2019 and 2020, as well as the incidence rate ratio.

**Results:**

During March and April there was a decrease in PED visits from 4,588 visits in 2019 to 2,527 visits in 2020 (ratio = .551, 95%CI [.52,.58]. Despite the drop in PED visits, the rate of hospitalizations rose with respect to 2019 (Risk Ratio = 1.31, p < .001, 95%CI [1.17,1.47]). Similar but more moderate trends were seen in the adult ED. From May-July 2020, after the lockdown was lifted, PED visits remained 30% below the same time period from 2018 and 2019, while the hospitalization rate returned to its pre-pandemic level.

**Conclusions:**

A significant drop in PED visits is seen to extend well beyond the peak of the pandemic and the lockdown period. This highlights the potential risk of children with serious emergencies becoming casualties of the pandemic by their not being brought to medical attention. Efforts should be made to raise public awareness among parents and other caretakers of children regarding this matter.

## Introduction

The first case of COVID-19 in Israel was confirmed on February 21, 2020. The patient’s infection was contracted overseas. In attempt to stem the tide of contagion within the general population which followed, the Israeli government imposed a nationwide lockdown on March 12, one day after the WHO declared COVID-19 to be a pandemic [1]. All educational institutions, for children of all ages, were closed.

Thus far, worldwide experience with COVID-19 suggests that while it may be severe in adults it usually manifests as a minor illness in children [2–4]. Though Multisystem Inflammatory Syndrome associated with COVID-19 has been reported among children in various countries [5–8], this severe complication is rare.

Since the onset of the pandemic, an increase in morbidity from various illnesses among adults due to delay or avoidance in seeking medical care has been reported. Fear of contagion and barriers to transport to the medical facility imposed by regional lockdowns are among the contributing factors. This phenomenon has even been reported with life-threatening emergencies such as acute myocardial infarction [9], and malignancies [10]. It has been noted as well among Israeli children with appendicitis [11]. A survey in Germany recently revealed a rise in cases of diabetic ketoacidosis among children with long-standing diabetes [12]. An increase in deaths has also been reported among children whose presentation to the emergency department was delayed [13]. A recent national U.S. survey published by the Center for Disease Control indicated a significant decline in visits to emergency departments (ED) among adults, and an even steeper decline among children [14]. Similar findings were reported from large pediatric emergency departments in the US [15] and Europe [16,17].

The aim of this study was to identify changes in trends of pediatric ED visits and hospitalization rates in our medical center since the onset of the pandemic. This data may allow us to more accurately project the appropriate sizes of the medical and nursing staff necessary to continue providing optimal medical care to all children presenting to our emergency department in the era of COVID-19.

## Aims

The principal aim of this study was to assess the number of pediatric ED visits as well as the hospitalization rates of these visits among children presenting to SZMC since the onset of the COVID-19 pandemic as compared to similar time frames during previous years. Additional aims included:

1. To evaluate whether the number of ED visits and hospitalization rates varied with the changes in the degree of disease burden in the general population and the lockdown status in the country.
2. To compare pediatric emergency department visits and hospitalization rates with that of the adult emergency department in our institution during the same time periods.
3. To assess how prolonged are the effects of COVID-19 on ED visits and hospitalization rates and how best to adapt to these changes.

## Materials and methods

The pediatric ED of SZMC serves Jerusalem and the surrounding areas in which reside roughly 500,000 children under the age of 18 years and provides care to approximately 35,000 children per year.

Data for this study were drawn from the SZMC computerized databank. The daily number of PED and adult ER visits as well as hospitalizations resulting from these visits during the months January-July of 2018, 2019, 2020 were recorded. This data was compared between three time periods: March-July 2019, March-July 2020, and May-July 2020. Data analysis was performed using SPSS v.25 (Aurora, NY). We compared the risk ratio (RR) for hospitalization the two years as well as the Incidence rate ratio (IRR). PED and adult ER visits for all three years were analyzed by the Poisson distribution within a Generalized Linear Modeling (GLM) followed by a monthly pairwise comparison (simple main effect; with Bonferroni correction for multiple comparison. Marginal means are plotted with uncorrected 95% Confidence Intervals as error bars. Monthly comparisons were calculated across years (year comparisons within each month) based on an IRR of hospitalizations to PED and adult ER visits between years. A logistic transformation was applied to the monthly comparisons in a similar form. Each plot was complemented by a table of significance for those months in which counts or percentages were different and the percent point change, i.e. the net change in year 2020 compared to years 2018 and 2019 (e.g., N2018/N2020-1)*100.

This study was approved by the institutional review board of SZMC (0134-20-SZMC).

## Results

**Fig 1:**
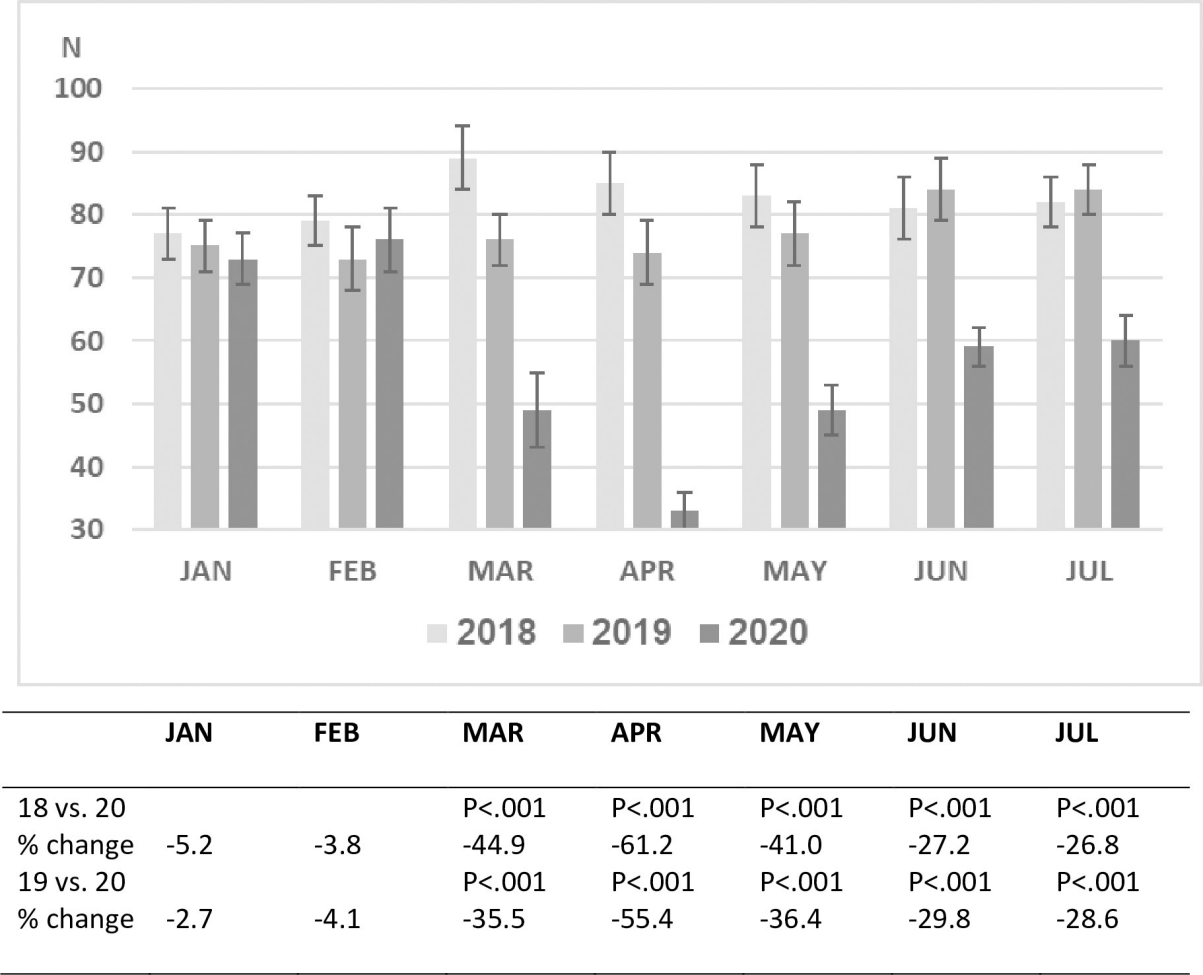
Mean daily PED visits by month and year.

During the years 2018 and 2019 approximately 35,000 children between the ages 0–18 years were treated in the pediatric emergency department of SZMC. As expected, the patient volume per month varied over the course of this 24-month period. A comparison of 3 time periods, January and February 2020 (prior to the pandemic), March and April 2020 (peak months of the epidemic in Israel accompanied by lockdown), and May-July 2020 (months with a decrease in disease prevalence in Israel accompanied by the opening of schools and commerce), with the same time periods in 2018 and 2019 revealed that the number of pediatric ED visits and hospitalization rates during January and February of 2018, 2019 and 2020 were similar. A dramatic decline in ED visits (>50%) occurred in March and April of 2020, followed by a significant rise from May-July. Nevertheless, ED visits in July 2020 were 28% less than in July of the two preceding years (Fig 1).

**Fig 2:**
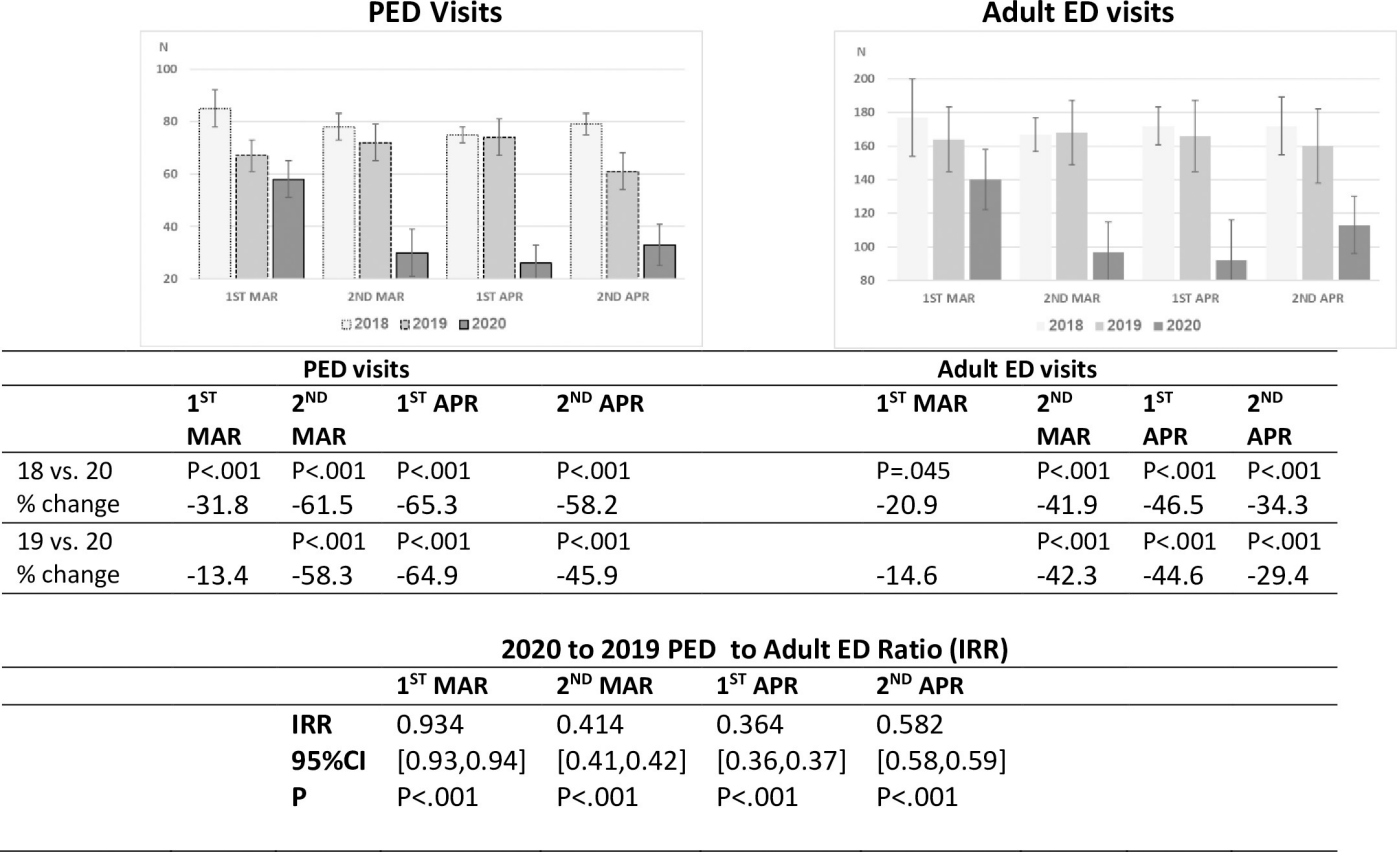
PED and adult ED mean daily visits, March and April 2018–2020, half-month periods. The decline in PED and adult ED mean daily visits started in the second half of March and continued significantly during the next three periods. The comparison of IRR for PED and adult ED visits between 2019 and 2020, shows that the decline in visits in the late three periods was higher in PED than in adult ED.

**Fig 3:**
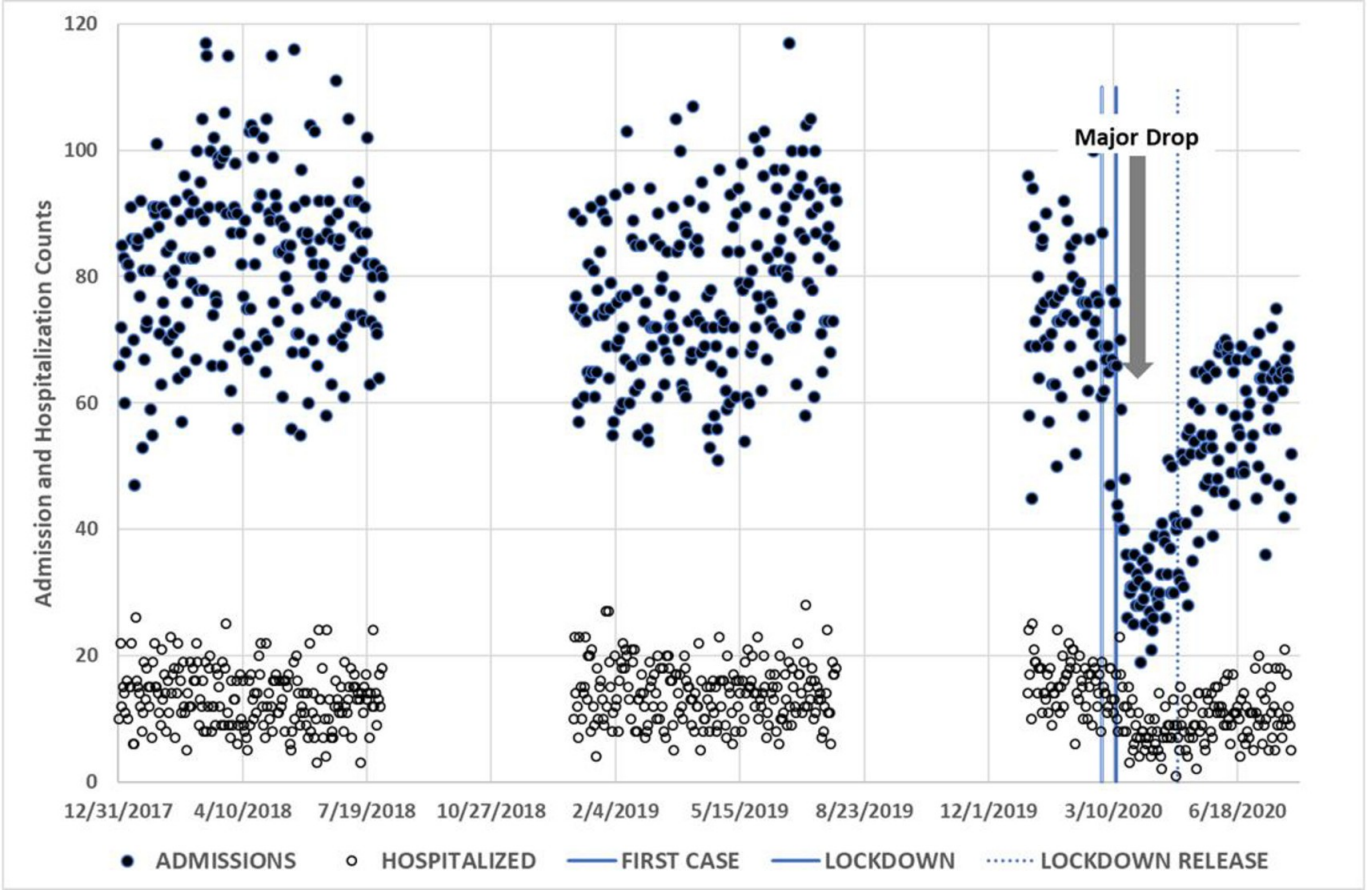
Plot of daily admission and hospitalization counts during 2018 to 2020. Vertical lines mark critical time points during the pandemic crisis: 1 March 2020, for first case discovered; 12 March 2020 for first lockdown; 1 May 2020 for lockdown release; Bold arrow shows a drop in PED admissions due to the pandemic.

**Fig 4:**
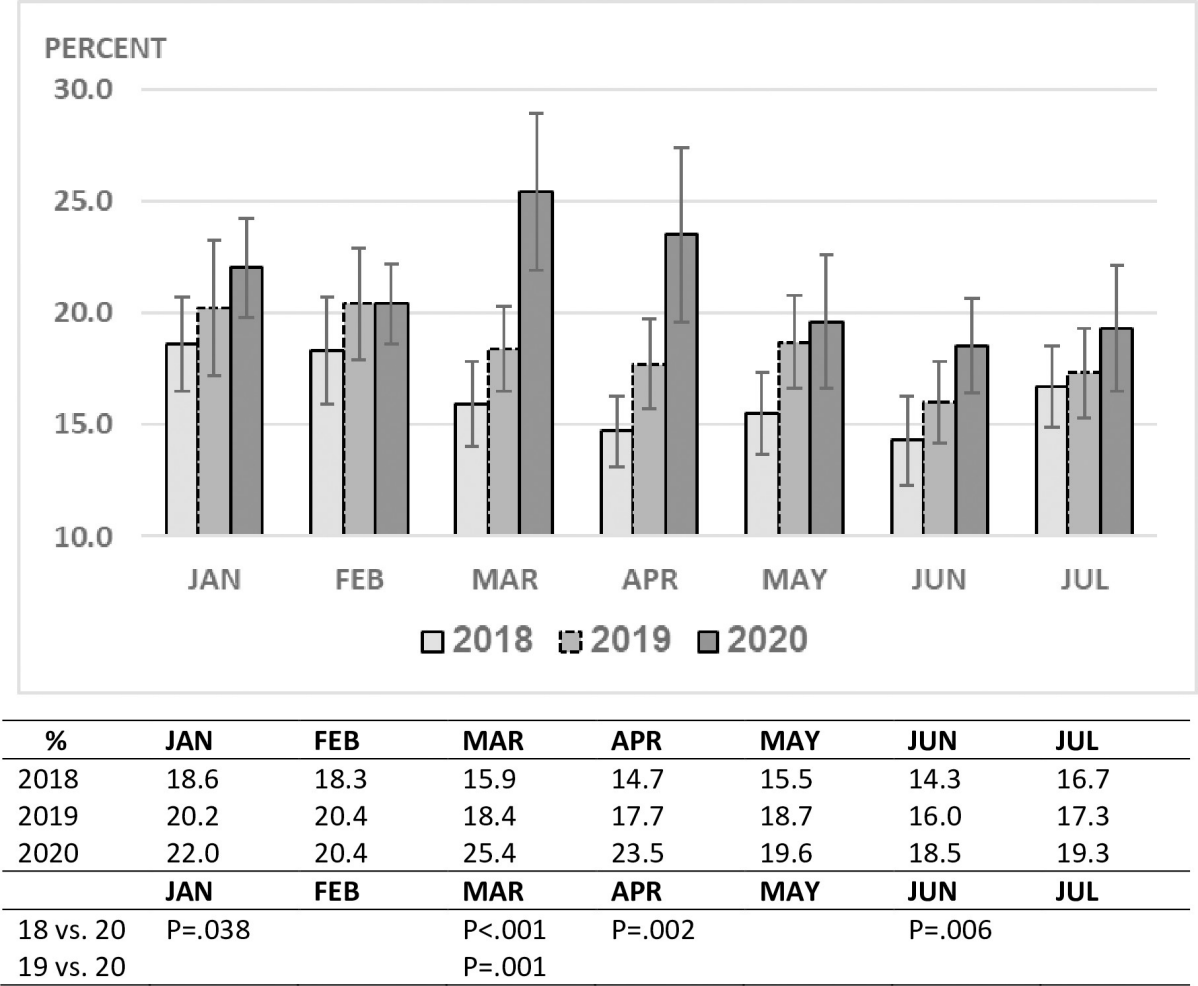
PED hospitalization rate by month and year.

Analysis of three specific developments during the first 5 months of the pandemic in Israel; the documented spread of COVID-19 in the general population (March 1^st^), the nationwide lockdown (March 12^th^) and the re-opening of schools (May 3^rd^) and their effects on ED visits and hospitalization rates in comparison to 2018–2019, revealed a slight decrease in ED visits during the first half of March followed by a maximum decline of over 60% during the lockdown period until the school system re-opened (Fig 2). The decline in ED visits correlated with an increase in hospitalization rates (Fig 3). This was most evident during the lockdown period when the rate was 23–25% as compared to 15–18% (p <0.001) during the same time frame of the previous two years (Fig 4). The rise in ED visits during the May-July period from the nadir of March-April to 30% below the previous years, was accompanied by a parallel decline in the hospitalization rate to 18–19%, close to the pre-pandemic level (Figs 1 and 2).

**Fig 5:**
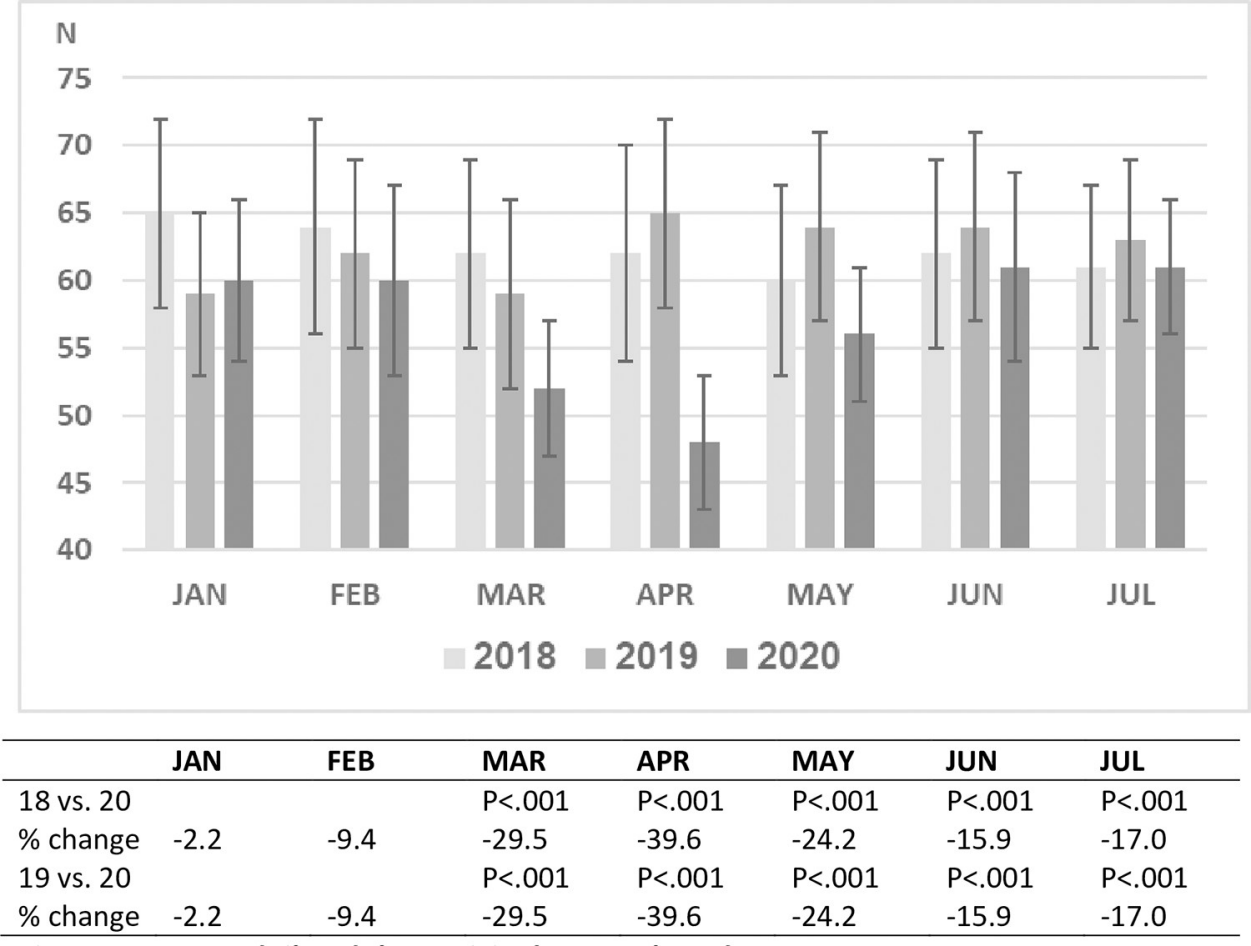
Mean daily adult ED visits by month and year.

**Fig 6:**
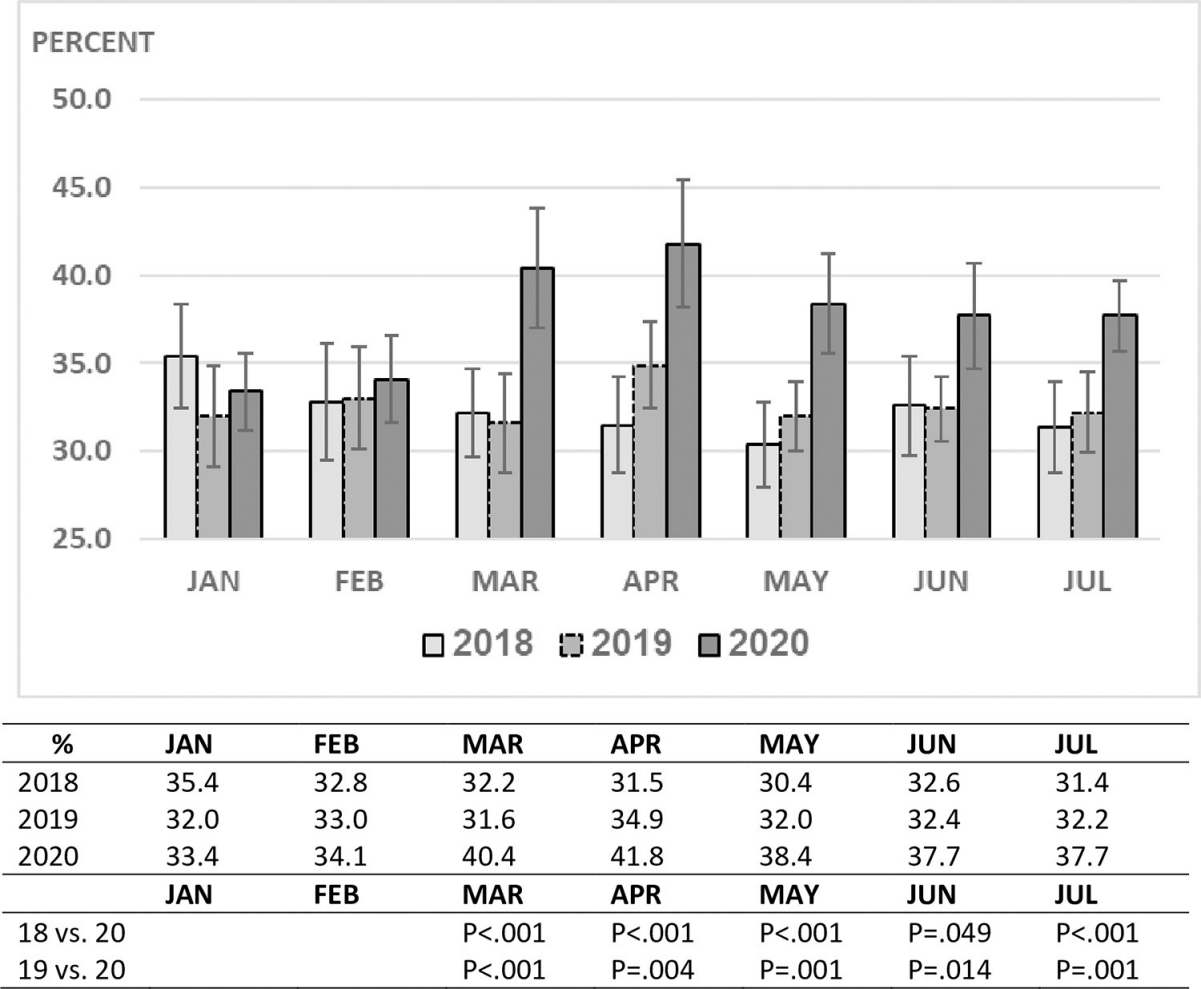
Adult ED hospitalization rate (%) by month and year.

A decrease in the number of ED visits during comparable time periods was observed as well in the adult ED of our medical center, though more moderate in magnitude. Adult ED visits during March and April declined by 30–40%, compared to 50–60% in pediatric ED visits (p< 0.001) (Fig 5). By July, adult ER visits rose to just 17% below July of the two previous years, in comparison to 28% in the pediatric ED (Fig 3). Hospitalization rates among adults before and after the pandemic began were 33% and 40%, respectively (p<0.001), similar to the trend we found in children (Fig 6).

Table 1 summarizes our data. Three time periods are represented: 1. March-July (the entire study period); 2. March-April (onset of pandemic and full lockdown period); 3. May-July (period during which restrictions were eased). A drop in pediatric ED visits is seen throughout the entire study period. The nadir occurred during March and April, with a significant IRR between 2019 and 2020. A concurrent rise in hospitalization rates is also observed (RR), which is less dramatic during the May-July period (P = 0.016). The drop in ED visits is greater among children than for adults (IRR) though the risk ratio is similar.

**Table 1:** Incidence rate ratio and risk ratio between 2019 and 2020; PED and adult ED.

			March-July			March-April			May-July	
	Year	C1	C2	Risk: C1/C2	C3	C4	Risk: C3/C4	C5	C6	Risk: C5/C6
		H	V		H	V		H	V	
**PED**	**R1:** 2019 Count	14 2,110	79 12,112	0.174 [0.17,0.18]	14 824	75 4,588	0.180 [0.17,0.19]	14 1,286	82 7,524	0.171 [0.16,0.18]
	**R2:** 2020 Count	10 1,564	50 7,690	0.203 [0.19,0.22]	10 594	40 2,527	0.235 [0.22,0.26]	11 970	56 5,163	0.188 [0.18,0.20]
	**IRR: R2/R1**	0.741 [0.69,0.79]	0.635 [0.62,0.65]	**Risk Ratio** 1.17, p < .001 [1.09,1.25]	0.721 [0.65,0.80]	0.551 [0.52,0.58]	**Risk Ratio** 1.31, p < .001 [1.17,1.47]	0.754 [0.69,0.83]	0.686 [0.66,0.72]	**Risk Ratio** 1.10, p = .016 [1.00,1.20]
**Adult ED**	**R3:** 2019 Count	63 9,651	191 29,224	0.330 [0.32,0.34]	62 3,790	185 11,261	0.337 [0.32,0.35]	64 5,861	195 17,963	0.326 [0.32,0.34]
	**R4:** 2020 Count	56 8,556	144 21,972	0.389 [0.38,0.40]	50 3,077	125 7,647	0.402 [0.39,0.42]	60 5,479	156 14,325	0.382 [0.37,0.40]
	**IRR: R3/R4**	0.886 [0.86,0.91]	0.752 [0.74,0.77]	**Risk Ratio** 1.18, p < .001 [1.14,1.22]	0.812 [0.77,0.85]	0.679 [0.66,0.70]	**Risk Ratio** 1.20, p < .001 [1.13,1.26]	0.935 [0.90,0.97]	0.797 [0.78,0.82]	**Risk Ratio** 1.17, p < .001 [1.12,1.22]
	**Risk R**_**child**_**/Risk R**_**adult**_			0.94, p = .890 [0.85,1.04]			1.10, p = .075 [0.96,1.25]			0.99, p = .598 [0.91,1.07]

C1-C6: Daily mean and total count; In squared brackets, 95% confidence interval; H = Hospitalization, V = ED visits; C1/C2, C3/C4 and C5/C6 = Percentage of daily hospitalizations (RISK percent); IRR = Incidence rate Ratio; Risk Ratio = Risk_2020_/Risk_S2019_; R2/R1 and R3/R4 are the ratio between daily mean for 2019 and 2020 of PED and adult ED, respectively. Daily mean was calculated taking two decimal points.

## Discussion

In this study we observed a steep decline in our pediatric ED visits since the COVID-19 pandemic began, a finding reported by other pediatric EDs in different parts of the world. Fear of contagion with the coronavirus, a reduction in prevalence of other infectious diseases due to infection control barriers imposed because of COVID-19 [18–20], adaptive changes in HMO practices which then generate less ED referrals and an increased emphasis and availability of telemedicine as a diagnostic tool [21–23], all are likely contributors to this phenomenon.

In light of this, a critical question to be asked is whether or not the decline in ED visits applies as well to patients whose medical or surgical problems are potential life-threatening emergencies. Wise [13] reported cases of death resulting from avoidance of seeking medical care since the onset of the pandemic. Two different medical centers observed that the percentage of severely ill patients presenting to the PED had risen [17,24].

We found significant differences in the changes in ED visits and hospitalization rates between the pediatric and adult departments since the onset of the pandemic. A 30–40% decrease in adult ED visits occurred during March and April as compared to 40–60% in the pediatric ED. (P<0.001) This likely reflects the difference in the nature of the medical problems which present at either site. As children more commonly suffer from infectious diseases, the various protective barriers imposed to control the COVID-19 outbreak likely reduce the spread of other communicable infections. Additionally, since the coronavirus causes more significant disease in adults [25], the number of ED visits for COVID-19 related illness among adults was substantially greater than for children who only rarely presented to the ED because of COVID-19. During the months March-July 2020 approximately 3000 COVID-19 PCR tests, 20% of which were positive, were performed in the adult ED as opposed to 150 and 7% respectively, in the pediatric ED.

Parallel to the marked drop in pediatric ED visits during March and April 2020, the hospitalization rate increased. This was likely due to less children with mild illnesses being brought to the ED and perhaps as well due to a delay in bringing children during this period to the emergency room which resulted in some of them presenting in a more advanced stage of their illness.

This study’s focus was to understand the effects of the COVID-19 epidemic on the number of our pediatric ED visits as well as on hospitalization rates. Three months after the ease of many restrictions and the re-opening of schools, the patient volume in our department remained 28% less than that of prior years. After rising to 25% at the peak of the pandemic here, the hospitalization rate from our PED dropped to 18% during the following 3-month period, close to the pre-COVID-19 rate. This suggests that the distribution of disease severity among children presenting to the ED has now begun to return to the pre-COVID-19 pattern. These particular phenomena have not been previously reported. Increased hospitalization rates among children during the COVID-19 pandemic was reported in Philadelphia [24] and Italy [15,17].

The yearly number of children treated at the PED of SZMC accounts for greater than 50% of all PED visits in Jerusalem. Approximately 100 children are seen daily in our PED. In addition to its retrospective nature therefore, another limitation of this study is that its findings may not reflect the realities of different regions of Israel nor those of other countries. Varying access to HMO care in the community and varied policies of different HMOs regarding indications and insurance coverage for ED visits may alter regional ED visit patterns. Similarly, non-uniformity in access to care via telemedicine between different regions would also contribute to varying trends in ED visits [21]. Lastly, as the prevalence of COVID-19 in any given community changes with time, our findings can only teach us what was, but not what lies ahead.

Limitations of the electronic data system of our medical center and the retrospective design of this study did not allow us to compare the level of disease severity among children presenting to the ED before and after the pandemic began. The hospitalization rate of ED visits was instead used as a surrogate marker.

COVID-19 has profound effects, both direct and indirect, on child healthcare. Among the indirect, of chief concern is that children with serious emergencies not become casualties of the pandemic by their not being brought to medical attention. Efforts should be made to raise public awareness among parents and other caretakers of children regarding this matter. Further, prospective studies would aid in understanding the scope of this phenomenon. As pediatric EDs grapple with new issues relating to triage and patient isolation, among others, it would also be important to specifically determine for which medical conditions there now are more ED visits, and for which there are less. As the pandemic continues, necessary resources including human, technical, financial, and medical uniquely relevant to each pediatric ED should be calculated and made available to meet the various challenges which these times present.

## Supporting information

S1 Dataset (XLSX)

## References

[1] Availale: https://www.who.int/dg.speeches/detail/who-director-general—s-opening-remarks-at-the-media-briefing-on-covid-19-11-march-2020 [accessed 1 April 2020].

[2] Ludvigsson JF. Systematic review of COVID-19 in children shows milder cases and better prognosis than adults. Acta Paediatr.2020; 109: 1089–1095. 10.1111/apa.15270 32202343 PMC7228328

[3] Brodin P. Why is COVID-19 so mild in children? Acta Paediatr. 2020;109:1082–1083. 10.1111/apa.15271 32212348

[4] Livingston E. Bucher K. Coronavirus disease 2019 (COVID-19) in Italy. JAMA 2020; 323:1335. 10.1001/jama.2020.4344 32181795

[5] Riphogen S. Gomez X. Gonzales-Martinez C. et al,. Hyperinflammatory shock in children during COVID-19 pandemic. Lancet 2020; 395:1607–1608. 10.1016/S0140-6736(20)31094-1 32386565 PMC7204765

[6] Whittaker E. et al, for the PIMS-TS Study Group and EUCLIDS and PERFORM Consortia. Clinical Characteristics of 58 Children With a Pediatric Inflammatory Multisystem Syndrome Temporally Associated With SARS-CoV-2. JAMA 2020; 324: 259–269. 10.1001/jama.2020.10369 32511692 PMC7281356

[7] Feldstein LR. et al, For the Overcoming COVID-19 Investigators and the CDC COVID-19 Response Team. Multisystem Inflammatory Syndrome in U.S. Children and Adolescents. N Engl Med 2020; 383: 334–346. 10.1056/NEJMoa2021680 32598831 PMC7346765

[8] Castagnoli R. et al. Severe Acute Respiratory Syndrome coronavirus 2 (SARS-CoV-2) Infection in Children and Adolescents, A Systematic Review. JAMA Pediatr.2020; 174:882–889. 10.1001/jamapediatrics.2020.1467 32320004

[9] Zylke JW, Bauchner H. Mortality and morbidity, The measure of a Pandemic. JAMA 2020;324:458–459. 10.1001/jama.2020.11761 32609308

[10] Kaufman HW, Chen Z, Niles J, Fesko Y. Changes in the Number of US Patients With Newly Identified Cancer Before and During the Coronavirus Disease 2019 (COVID-19) Pandemic. JAMA Network Open. 20920;3: e2017267. 10.1001/jamanetworkopen.2020.17267 32749465 PMC7403918

[11] Snapiri O, Rosenberg Danziger C, Krause I, et al. Delayed diagnosis of pediatric appendecitis during the COVID-19 pandemic. Acta Paediatr. 2020; 109: 1672–1676. 10.1111/apa.15376 32460364 PMC7283758

[12] Kamrath C, Monkemoller K, Biester TR. et al. Ketoacidosis in Children and Adolescents With Newly Diagnosed of Type 1 Diabetes during the COVID-19 Pandemic in Germany. JAMA. 2020; 324: 801–804. 10.1001/jama.2020.13445 32702751 PMC7372511

[13] Wise J. Covid-19: delays in attending emergency departments may have contributed to death 0f nine children. BMJ. 202;369: m2624. 10.1136/bmj.m2624 32606039

[14] Hartnett KP, Kite-Powell A, De Vies J, et al. Impact of the COVID-19 Pandemic on Emergency Department Visits_ united States, January 1,2019- May 30, 2020. MMWR 2020; 69;699–704. 10.15585/mmwr.mm6923e1 32525856 PMC7315789

[15] Cozzi G, Zanchi C, Giangreco M, et al. the impact of the COVID-19 Lockdown in Italy on a paediatric emergency setting. Acta Paediatrica. 2020;109:2157–2159. 10.1111/apa.15454 32598519 PMC7361857

[16] Isba R, Edge R, Jenner R, et al. where have all the children gone? Decreases in paediatric emergency department attendances at the start of the COVID-19 pandemic of 2020. Arch Dis Child 2020; 105; 704. 10.1136/archdischild-2020-319385 32376695

[17] Scaramuzza A, Tagliaferri F, bonetti L, et al. changing admission patterns in paediatric emergency departments during the COVID-19 pandemic. Arch Dis Child 2020; 105:704–706. 10.1136/archdischild-2020-319397 32461213

[18] Donohue JM, Miller E. COVID-19 and School Closure. JAMA 2020; 324:845–847. 10.1001/jama.2020.13092 32745182

[19] Auger KA, Shah SS, Richardson T, et al. Association between Statewide School Closure and COVID-19 Incidence and Mortality in the US. JAMA 2020; 324:859–870. 10.1001/jama.2020.14348 32745200 PMC7391181

[20] Yehya N, Venkataramani A, Harhay MO. Statewide Intervention and COVID-19 mortality in the United States: An Observational Study. Clin Infect Dis 2020. 10.1093/cid/ciaa923 32634828 PMC7454446

[21] Portnoy J, Waller M, Elliot T. Telemedicine in the Era of COVID-19. J Allergy Clin Immunol Pract 2020; 8: 1489–1491. 10.1016/j.jaip.2020.03.008 32220575 PMC7104202

[22] Gamus A, Chodic G. Telemedicine after COVID-19: The Israeli Perspective. IMAJ 2020;22: 401–403. 33236577

[23] Grossman Z. Chodick D, Reingold SM. Chapnic G, Ashkenazi S. The future of telemedicine visits after COVID-19: perception of primary care pediatricians. Isr J Health Poli Research 2020; 9:53 10.1186/s13584-020-00414-0 PMC7573530 33081834

[24] Chaiyachati BH, Agawu A, Zorc JJ, Balamuth F. Trends in Pediatric Emergency Department Utilization after Institution of Coronavirus Disease-19 Mandatory Social Distancing. J Pediatr 202: 1–4. 10.1016/j.jpeds.2020.07.048 32702427 PMC7370904

[25] Ludvigsson JF. Systematic review of COVID-19 in children shows milder cases and a better prognosis than adults. Acta Paediatr. 2020;109:1088–1095. 10.1111/apa.15270 32202343 PMC7228328

